# Chiral Grayscale Imaging Based on a Versatile Metasurface of Spin-Selective Manipulation

**DOI:** 10.3390/ma18133190

**Published:** 2025-07-05

**Authors:** Yue Cao, Yi-Fei Sun, Zi-Yang Zhu, Qian-Wen Luo, Bo-Xiong Zhang, Xiao-Wei Sun, Ting Song

**Affiliations:** School of Mathematics and Physics, Lanzhou Jiaotong University, Lanzhou 730070, China

**Keywords:** metasurface, chiral grayscale imaging, high resolution, spin-selective manipulation

## Abstract

Metasurface display, a kind of unique imaging technique with subwavelength scale, plays a key role in data storage, information processing, and optical imaging due to the superior performance of high resolution, miniaturization, and integration. Recent works about grayscale imaging as a typical metasurface display have showcased an excellent performance for optical integrated devices in the near field. However, chiral grayscale imaging has been rarely elucidated, especially using a single structure. Here, a novel method is proposed to display a continuously chiral grayscale imaging that is adjusted by a metasurface consisting of a single chiral structure with optimized geometric parameters. The simulation results show that the incident light can be nearly converted into its cross-polarized reflection when the chiral structural variable parameters are *α* = 80° and *β* = 45°. The versatile metasurface can arbitrarily and independently realize the spin-selective manipulation of wavelength and amplitude of circularly polarized light. Due to the excellent manipulation ability of the versatile metasurface, a kind of circularly polarized light detection and a two-channel encoded display with different operating wavelengths are presented. More importantly, this versatile metasurface can also be used to show high-resolution chiral grayscale imaging, which distinguishes it from the results of previous grayscale imaging studies about linearly polarized incident illumination. The proposed versatile metasurface of spin-selective manipulation, with the advantages of high resolution, large capacity, and monolithic integration, provides a novel way for polarization detection, optical display, information storage, and other relevant fields.

## 1. Introduction

Optical metasurfaces, composed of two-dimensional arrays of periodic nanostructures arranged on a compact and planar substrate, can arbitrarily manipulate the various fundamental properties of the incident light at the nanometer scale since different meta-atoms can be utilized for manipulating the corresponding amplitude [1,2], phase [3,4,5,6], wavelength [7,8,9], and polarization states [10,11,12]. Depending on their excellent capability, metasurfaces are used to design varieties of novel meta-devices, such as metalenses [13,14], holograms [15,16,17], grayscale imaging [18,19], and vortex generators [20,21,22]. Recently, metasurface imaging has become an efficient and emerging approach to store and transmit optical information, which is demonstrated by plentiful research. Unlike complicated optical holograms that are recorded interference patterns in the far field, grayscale imaging only contains the amplitude profile, resulting in the image processing algorithm and manufacturing operation of the metasurface being uncomplicated [23,24,25], which has attracted broad attention owing to its extensive application prospects in metasurface display. During the grayscale imaging encoding process, different nanostructures of the metasurface are spatially arranged as image pixels, which possess different output intensities. One straightforward scheme to realize continuous grayscale imaging with high resolution is to employ a series of nanostructures that can arbitrarily manipulate light intensity to encode the image. For example, Fuyong Yue et al. [26] proposed a metasurface that can exhibit a high-resolution grayscale image in the reflective laser beam with a spatially variant amplitude profile, which demonstrates that the grayscale imaging technology provides a new opportunity for metasurface display. Subsequently, Zheng’s group showed an anti-counterfeiting metasurface that can simultaneously realize a two-channel grayscale imaging in the near field [23], which has been a hot research topic. Although a series of studies on grayscale imaging has been produced in recent years, the chiral grayscale imaging of metasurface using planar meta-atoms has yet to be reported to the best of our knowledge.

Chirality, a geometrical property that prevents an object from being superimposed onto its mirror image, translates to a peculiar optical response, in which cross-coupling between the electric and magnetic fields of different circular polarizations (CPs) propagating in the chiral medium exists because there are no symmetric planes. Chiral structures can be effortlessly found in natural materials [27,28], such as mineral crystals [29,30,31], organic macromolecules [29,32], DNA [33,34], etc. However, the chirality of natural chiral materials is comparatively weak because a significant thickness of natural chiral structure is required in order for the material to be sufficiently cumulative for effective applications. Hence, optical devices composed of natural chiral materials lack integration and minimization; therefore, metasurfaces provide new opportunities to circumvent this drawback and break through the limitations of natural materials. Chiral metasurfaces can be used in various promising applications in many fields, such as optical communications, quantum computing, and biosensing, which benefit from the excellent performance of electromagnetic regulation. In particular, Zhancheng Li et al. [35] designed a chiral mirror that could realize the full-dimensional independent manipulation of circular polarized waves, which inspired us to design a metasurface for displaying continuous chiral grayscale imaging. In recent years, continuous grayscale imaging approaches based on metamaterials and metasurfaces have drawn sufficient attention, benefiting from rapidly changing nanofabrication techniques [36,37], whereas most of the existing chiral imaging approaches can only display binary images with two colors of black and white since the pixels possess different output intensities under left circularly polarized (LCP) and right circularly polarized (RCP) illumination, in which the information capacity and image resolution are also limited. At present, the prevalent grayscale imaging metasurfaces are composed of a single cell regarded as a polarizer [38,39,40,41], which can distinctly reveal one continuous grayscale image and even multiple grayscale images under the linearly polarized beam in the near field. However, the chiral grayscale imaging metasurfaces are rarely reported. Thus, it is highly desirable to design a chiral imaging metasurface, especially continuous grayscale imaging, for the potential application in optical imaging and biosensing.

In this work, we propose a versatile metasurface consisting of a chiral structure that demonstrates a high spin-selective manipulation by changing the relevant geometric parameters of the structure. Interestingly, the simulation results show that the amplitude of circularly polarized light can be arbitrarily adjusted. Based on the excellent capacity of the versatile metasurface, a circularly polarized light detection and a two-channel digital coding display are realized. In addition, a continuous chiral grayscale imaging is presented, which means our approach reveals a new strategy for high-resolution imaging different from previous grayscale imaging of linearly polarized light. This versatile metasurface, with efficient spin-selective manipulation and high resolution, provides an efficient platform for various future applications in polarization detection, image encryption, chiroptical sensing, and information storage.

## 2. Materials and Methods

**Figure 1:**
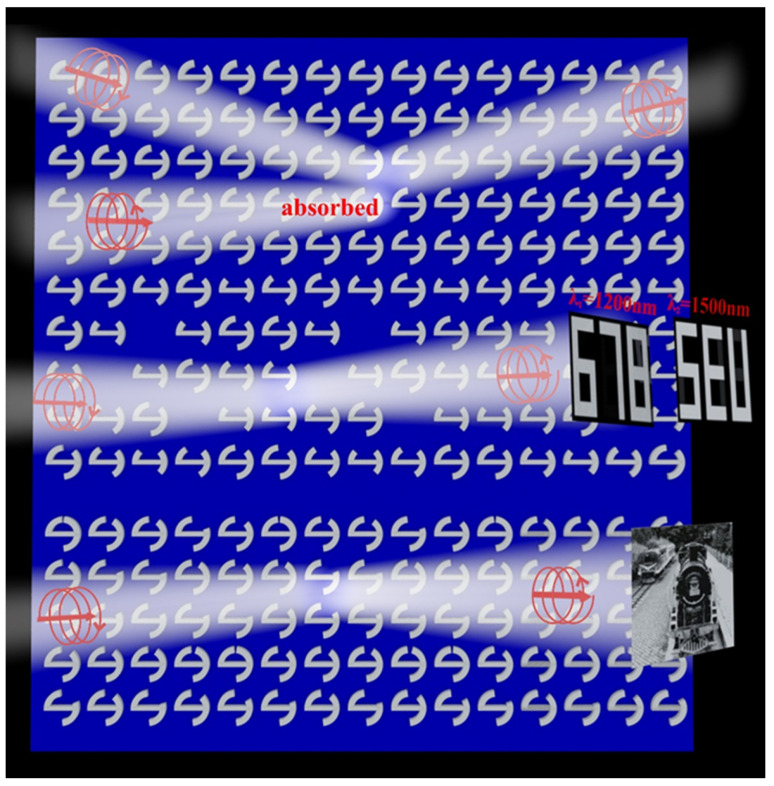
Schematic of the versatile metasurface that can perfectly realize circularly polarized detection, two-channel encoded display, and high-resolution chiral grayscale imaging.

A versatile metasurface composed of chiral structures that have high spin-selective manipulation is shown in Figure 1, which can be used as a kind of circularly polarized light detection, a two-channel encoding display, and continuous chiral grayscale imaging with high resolution. The proposed metasurface is a metal–insulator–metal (MIM) design, which is formed from a ground aluminum (Al) layer, a silicon dioxide (SiO_2_) spacer layer, and a top layer of aluminum chiral structures. Here, the unit cell of the versatile metasurface consists of a kind of chiral structure with a pair of symmetrical long arcs, a short arc, and a slender rod. As shown in the scheme, the unit cells contain three segmented sub-arrays, which can manipulate and generate different functions by adjusting the long arc and the short arc. In a word, the proposed versatile metasurface can reflect RCP waves if the incident light is LCP, while absorbing LCP waves with polarization change. Furthermore, it demonstrates that two images can hide in the LCP reflected beam under the presupposed wavelengths at 1200 nm and 1500 nm. More importantly, the versatile metasurface also exhibits continuous chiral grayscale imaging with high resolution under RCP illumination with polarization change. Hence, our proposed metasurface realizes the spin-selective multidimensional manipulation of optical waves and presents a novel approach for continuous chiral grayscale imaging.

**Figure 2:**
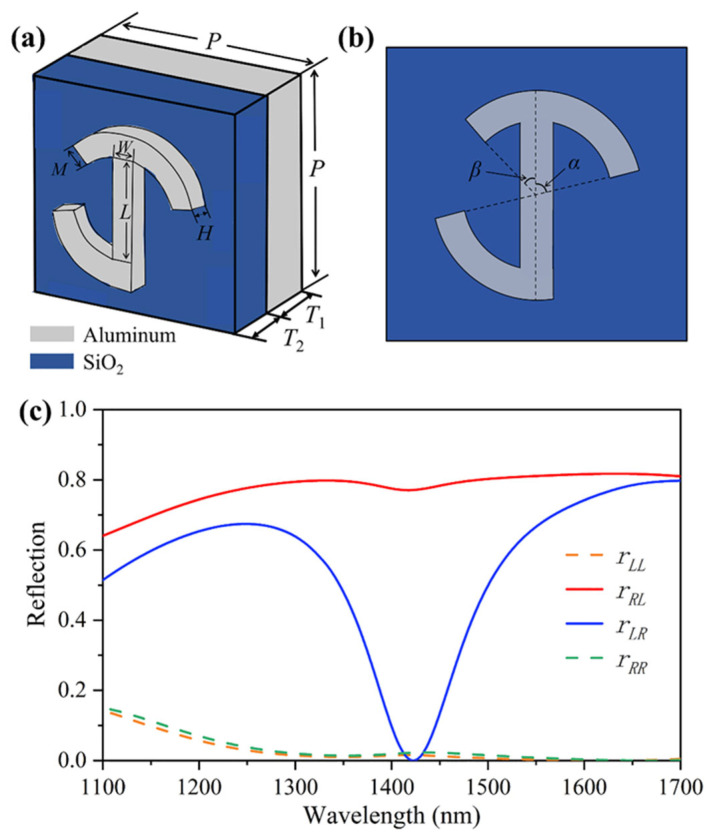
Schematics of chiral structures and simulated reflection spectra. (**a**,**b**) Schematic of the unit cells of different perspectives on a ground aluminum substrate and a SiO_2_ spacer layer. The chiral structure consists of a nanorod, a short arc, and a pair of symmetrical long arcs. (**c**) Simulated reflection spectra of the proposed chiral metasurface under circular polarization illumination.

The versatile metasurface consists of the proposed chiral structures shown in the schematic in Figure 2a,b. The chiral structures are a MIM design including a ground aluminum layer, a SiO_2_ spacer layer, and a top layer of aluminum chiral structures, which have a pair of symmetrical long arcs, a short arc, and a slender rod. The periods of all metasurfaces are *P* = 650 nm, the thicknesses of the ground aluminum layer and the SiO_2_ spacer layer are *T*_1_ = 300 nm and *T*_2_ = 180 nm, respectively. The parameters of the proposed chiral structures are as follows: the slender rod has a length of *L* = 160 nm, width of *W* = 70 nm, the symmetrical long arc and short arc have a width of *M* = 70 nm, and the chiral structure has a uniform height of *H* = 70 nm, as shown in Figure 2a. When the parameters are precisely adjusted, the proposed chiral metasurface can realize the spin-selective reflection, the details and discussions are shown in Figure S1 of Supplementary Materials. Figure 2b is a top view of the structural variables in a unit cell of the chiral structure, where the angles of the symmetrical long arc and the short arc are marked as *α* and *β* with respect to the *y*-axis, respectively. It is interesting that the wavelength and amplitude of the incident wave can be independently manipulated by changing the above structural parameters (*α* and *β*), as shown in Figure S2 of Supplementary Materials. Figure 2c shows the simulated reflection spectra; the red and blue solid lines are the reflection intensities with polarization change (the subscript “L” and “R” express the polarization state of the reflected and incident waves, respectively, where the first capital letter is the polarization state of the reflected wave) for the proposed chiral structures with the structural variable parameters of *α* = 80° and *β* = 45°. The simulation results indicate that the incident light is converted into its cross-polarized reflection (*r_RL_* and *r_LR_*), and the co-polarized reflection (*r_LL_* and *r_RR_*) is already near zero in the wavelength range from 1300 to 1700 nm, the more details and discussions are shown in Sections S3 and S4 of Supplementary Materials. More importantly, the proposed chiral metasurface can realize an excellent capacity of spin-selective manipulation around 1400 nm. In addition, we study the effect of different incident angles for the reflection intensities, the simulation results demonstrate that the proposed chiral metasurface still remains an excellent spin-selective manipulation when the incident angle is less 30°, as shown in Figure S5 of Supplementary Materials.

## 3. Results

**Figure 3:**
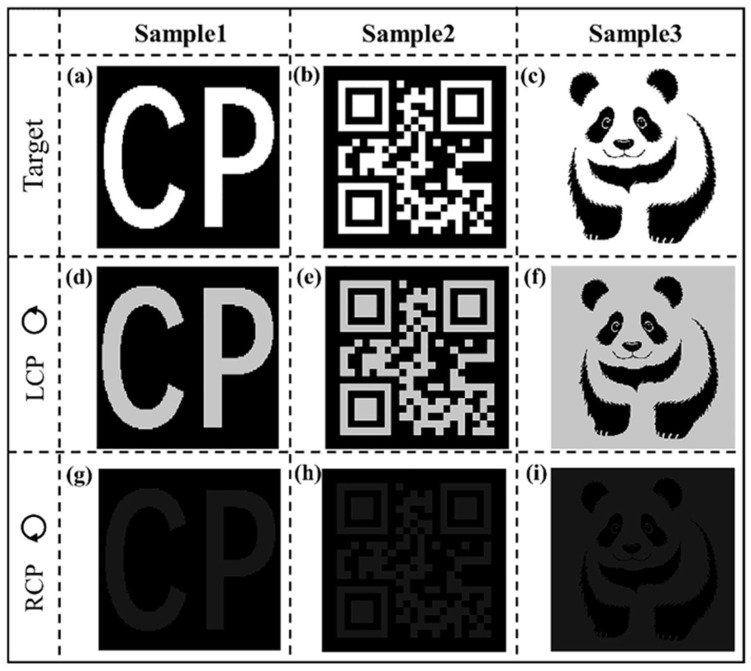
Simulation results of the proposed metasurface as a kind of circularly polarized detector. The first row presents the target pictures of different samples (**a**–**c**). The second (**d**–**f**) and third (**g**–**i**) rows show the simulation results of different samples under left circularly polarized (LCP) and right circularly polarized (RCP) illumination, respectively.

Due to the excellent spin-selective manipulation of the proposed chiral structures, we designed a kind of circularly polarized detector as shown in Figure 3. The simulation results for different samples demonstrate that the detected images are well consistent with the target images under the LCP incident illumination with the polarization change at 1400 nm. Nothing distinct was detected for all samples under the RCP incident illumination with polarization change, which demonstrates that the proposed circularly polarized detected metasurface can effectively measure the polarization states of circularly polarized light.

**Figure 4:**
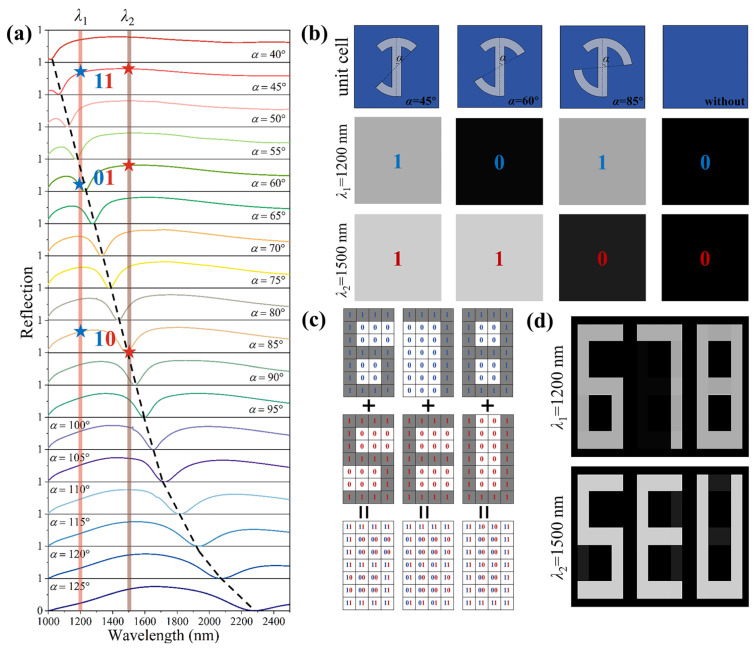
Schematics and simulated demonstration of the two-channel encoded display metasurface. (**a**) The simulated reflection spectra of the two-channel metasurface, which presents the principle of two-channel encoded display at 1200 nm and 1500 nm. (**b**) The schematics of unit cells of a two-channel encoded display metasurface. (**c**) The design approach of a two-channel encoded display metasurface. (**d**) The simulated results of a two-channel encoded display metasurface under RCP illumination at 1200 nm and 1500 nm, respectively.

Interestingly, we find that the operation wavelength can be effectively manipulated by adjusting the angle α; in other words, the spectral position of the global minimum of the reflection spectra of RCP illumination with polarization change can be tuned during 1000 nm to 2300 nm by varying the length of the long arc from α = 40° to 125°, as shown in Figure 4a. According to this characteristic, we designed a kind of wavelength-encoded display metasurface under the operating wavelengths of 1200 nm and 1500 nm, respectively. The principle of encoded display is shown in Figure 4b; it can choose three kinds of unit cells with different long arc lengths that correspond to the blue and red stars at λ_1_ = 1200 nm and λ_2_ = 1500 nm, as shown the reflection spectra in Figure 4a, whose angles α are 45°, 60°, and 85°, respectively, where the reflection intensity can be encoded as “1” and “0” depending on the operating wavelengths. It should be noted that the unit cells without structure are encoded as “0” and “0” under operating wavelengths. Figure 4c shows the encoded approach, which can present two switched grayscale images displayed by a single metasurface, including four coding patterns that correspond to four different unit cells. For example, each pixel of the first image of number “678” and another image of letter “SEU” can be marked as “1” and others marked as “0”, so the whole metasurface is formed from the coding patterns of “11”, “01”, “10”, and “00”. Figure 4d is the simulation result that presents two grayscale images of “678” and “SEU” under two wavelength illuminations of λ_1_ = 1200 nm and λ_2_ = 1500 nm, which are well in agreement with the design concept.

## 4. Discussion

**Figure 5:**
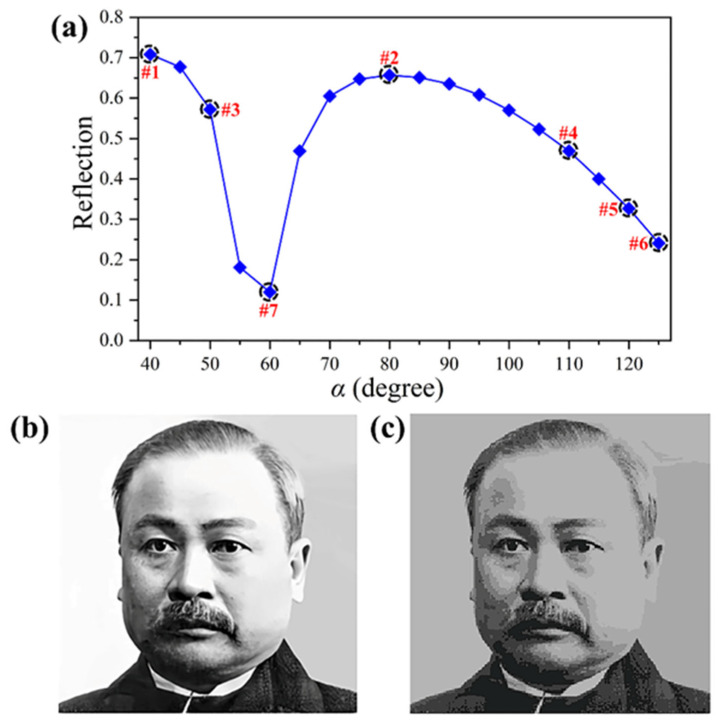
Schematics and simulation results of the chiral grayscale imaging metasurface. (**a**) The simulated reflected intensity for the chiral metasurface with the changing of *α* under RCP illumination at 1200 nm. (**b**,**c**) The target picture and simulation result of the chiral grayscale imaging metasurface. The grayscale images depict the portrait made for the sculpture of “Zhan Tianyou”.

In addition, we find that if the length of the long arc whose angle is marked as *α* has the appropriate adjustments, the reflection intensity can be manipulated accordingly. We chose seven structures, #1 to #7, with different *α*, as shown in Figure 5a. With these seven structures, we realized chiral grayscale imaging under RCP illumination with the polarization change at 1200 nm, where the object picture and simulation result of the chiral grayscale imaging metasurface are shown in Figure 5b,c, respectively. However, the maximum differences of reflection intensity between these seven structures are only 0.6, so the simulation result exhibits some discrepancies compared with the target picture due to the significant limitation of the imaging resolution.

**Figure 6:**
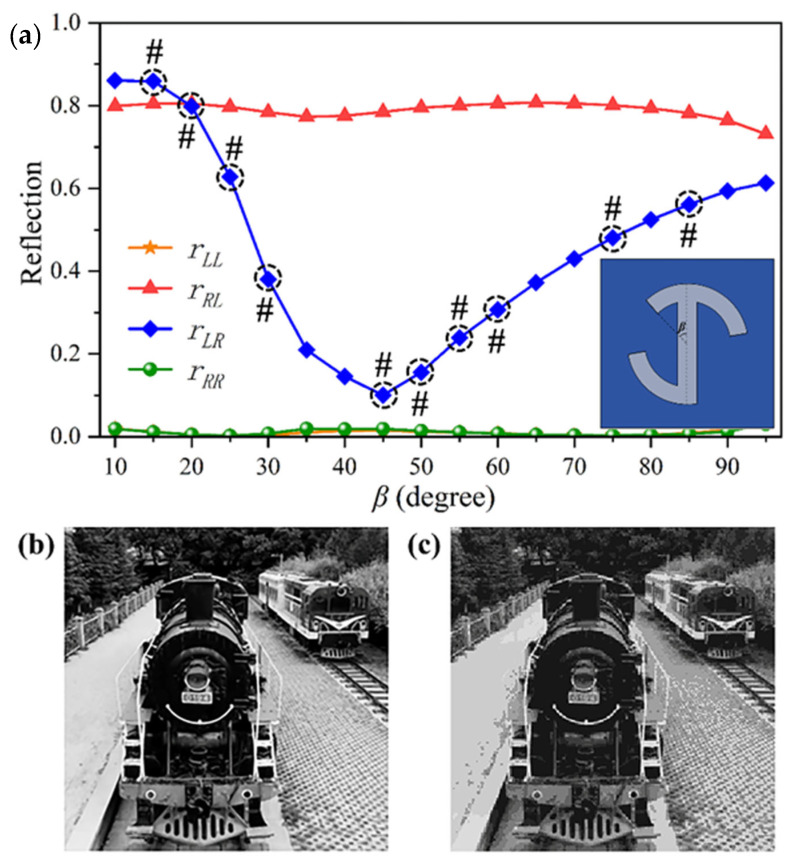
Schematics and simulation results of the continuous chiral grayscale imaging metasurface. (**a**) Simulated reflection spectra for the chiral metasurface with the changing of *β* under RCP illumination at 1400 nm, where the inset image is a schematic of the unit cell with angle *β*. (**b**,**c**) The target picture and simulation result of the continuous chiral grayscale imaging metasurface, where the target picture named “Locomotive Park” is from Lanzhou Jiaotong University.

Further, we adjust the length of the short arc whose angle is *β*; the result demonstrates that the amplitude of reflection can be arbitrarily manipulated by changing the length of the short arc under RCP illumination with the polarization change at 1400 nm, as shown in Figure 6a. The inset image is the top view of a schematic of a unit cell with angle *β*. According to this simulation result, there are ten structures with different lengths of short arcs that can be chosen to relatively uniformly manipulate the reflection intensity, whose lengths correspond to the angles *β* marked as #. Finally, we propose an efficient approach to realize a chiral grayscale imaging metasurface with high resolution by using the above ten structures. Here, we chose a target image of “Locomotive Park” belonging to Lanzhou Jiaotong University in Figure 6b. Figure 6c is the simulation result that agrees well with the target picture, which demonstrates that our proposed spin-selective metasurface can excellently display a high-resolution chiral grayscale imaging.

## 5. Conclusions

In conclusion, we designed and demonstrated a chiral grayscale imaging metasurface consisting of a single structure that can realize a high spin-selective manipulation of optical waves by changing the relevant geometric parameters of the structure. Meanwhile, we find that the proposed metasurfaces can simultaneously manipulate the operation wavelength and reflection intensity by adjusting the lengths of the long arc and short arc, respectively. Due to the excellent manipulation ability, we designed a circularly polarized light detection and a two-channel encoding display with different operating wavelengths. Further, we proposed a new approach to achieve a kind of continuous chiral grayscale imaging for circularly polarized light. The simulation results revealed that this strategy is different from previous grayscale imaging metasurfaces of linearly polarized light. Our work provides a more effective solution to realize high-resolution chiral grayscale imaging metasurfaces using a single structure, which provides a new way for image display, optical information storage, and polarized detection, which has applications in various fields.

## 6. Numerical Characterizations

All the reflection results were calculated by the computer simulation technology (CST) Microwave Studio software (CST Studio Suite 2020). The reflection intensities were calculated by the S-parameter of the 1D Result of CST, and the reflected signal polarization was confirmed by the Port Modes of the incident and reflection light.

## Data Availability

The original contributions presented in this study are included in the article. Further inquiries can be directed to the corresponding author.

## Supplementary Materials

The following supporting information can be downloaded at: https://www.mdpi.com/article/10.3390/ma18133190/s1, Figure S1: The effect of the structural parameter variation on the reflection intensity of the proposed chiral metasurface. The results are obtained with structural variables *α* = 80°, *β* = 45° and under the normal incident illumination. The reflection intensity of *r_RL_* and *r_lR_* (the cross-polarized components) of the proposed chiral metasurface with (a,b) changing *W*, (c,d) changing *H*, (e,f) changing *L*. Figure S2: The effect of the *α* and *β* variation on the reflection intensity of the proposed chiral metasurface. The results are obtained with structural variables *P* = 650 nm, *L* = 160 nm, *W* = 70 nm, *M* = 70 nm, *H* = 70 nm, *T*_1_ = 300 nm, and *T*_2_ = 180 nm under normal incident illumination. (a–d) The reflection intensity of *r_LL_* and *r_RR_* (the co-polarized components) of the proposed chiral metasurface with the changing of *α* and *β*. (e–h) The reflection intensity of *r_LR_* and *r_RL_* (the cross-polarized components) of the proposed chiral metasurface with the changing of *α* and *β*. Figure S3: Simulated absorption spectra under LCP and RCP illumination from 1100 to 1700 nm. Figure S4: The electric field distributions on the *xoy* section at the wavelength of 1420 nm under (a) LCP illumination, (b) RCP illumination. Figure S5: The effect of incident angle variation on the reflection intensity of the proposed chiral metasurface for different polarized components (a) is LL, (b) is RR, (c) is RL, (d) is LR.
