# Supplementary material for: Chiral Grayscale Imaging Based on a Versatile Metasurface of Spin-Selective Manipulation
Source: Materials (Basel). 2025 Jul 5;18(13):3190. doi: 10.3390/ma18133190 (PMC12250749; doi:10.3390/ma18133190)
Supplement: Supplementary file 1 [file materials-18-03190-s001.zip › materials-3665404-supplementary.pdf]

materials

MDPI

Supplementary Materials

Chiral Grayscale Imaging Based on a Versatile Metasurface of Spin-Selective Manipulation

Yue Cao [1,*], Yi-Fei Sun [1], Zi-Yang Zhu [1], Qian-Wen Luo [1], Bo-Xiong Zhang [1], Xiao-Wei Sun [1] and Ting Song [1]

Section S1. The effect of the structural parameter variation on the reflection intensity of the proposed chiral metasurface.

To validate the effect of the structural parameter variation on the reflection intensity of the proposed chiral metasurface, we simulate the reflection intensity of the cross-polarized components for the proposed chiral metasurface with the changing structural parameter, as shown in Figure S1. The simulated results demonstrate that with changing structural parameters, the value of r_{RL} is high and no significant changes occurred, as shown in **Figure S1 (a, c, e)**. With the changing of W and H, the value of r_{LR} has maximum and minimum, and there was no significant change for the global minimum value of the r_{LR}, which primarily distributes around 1500 nm, as shown in **Figure S1 (b, d)**. In addition, with the changing of L, the global minimum value of the r_{LR} shifts to the shorter wavelength band, as shown in **Figure S1 (f)**.

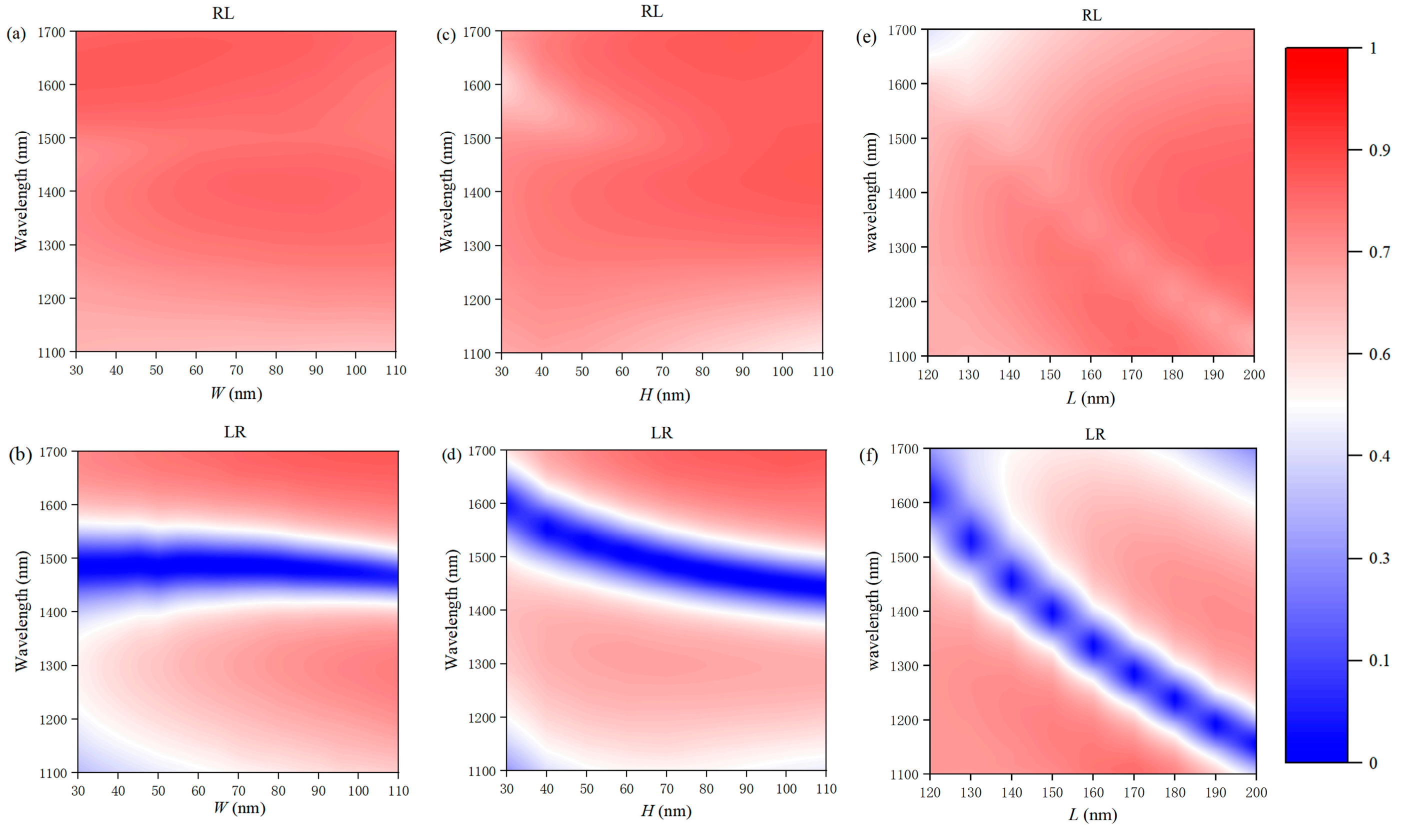

Figure S1. The effect of the structural parameter variation on the reflection intensity of the proposed chiral metasurface. The results are obtained with structural variables α=80°, β=45° and under the normal incident illumination. The reflection intensity of r_{RL} and r_{IR} (the cross-polarized components) of the proposed chiral metasurface with (a, b) changing W, (c, d) changing H, (e, f) changing L.

Section S2. The effect of the α and β variation on the reflection intensity of the proposed chiral metasurface.

 https://doi.org/10.3390/ma18133190

To show the effect of the α and β variation on the reflection intensity of the proposed chiral metasurface, we simulate the reflection intensity of the co-polarized and cross-polarized components for the proposed chiral metasurface with the changing α and β, as shown in **Figure S2**. The simulated results demonstrate that the reflection intensities of the co-polarized components are near close to zero when the angle of the long arc is less than 110°, as shown in **Figure S2 (a-d)**. On the contrary, the value of r_{LR} is high when the angle of the long arc is less than 100° and the short arc is less than 75°, as shown in **Figure S2 (e, g)**. In particular, as the angle of the long arc increases, the global minimum of the reflection shifts to the longer wavelength band, which well verifies that the wavelength of the incident wave can be manipulated by changing the angle of the long arc, as shown in **Figure S2 (f)**. From **Figure S2 (h),** we can find that the amplitude of RCP reflected waves (cross-polarized components) can be manipulated by changing the angle of the short arc at operation wavelength. As mentioned above, the proposed chiral structures with suitable angles of long arc α=80° and short arc β=45°present excellent spin-selective manipulation.

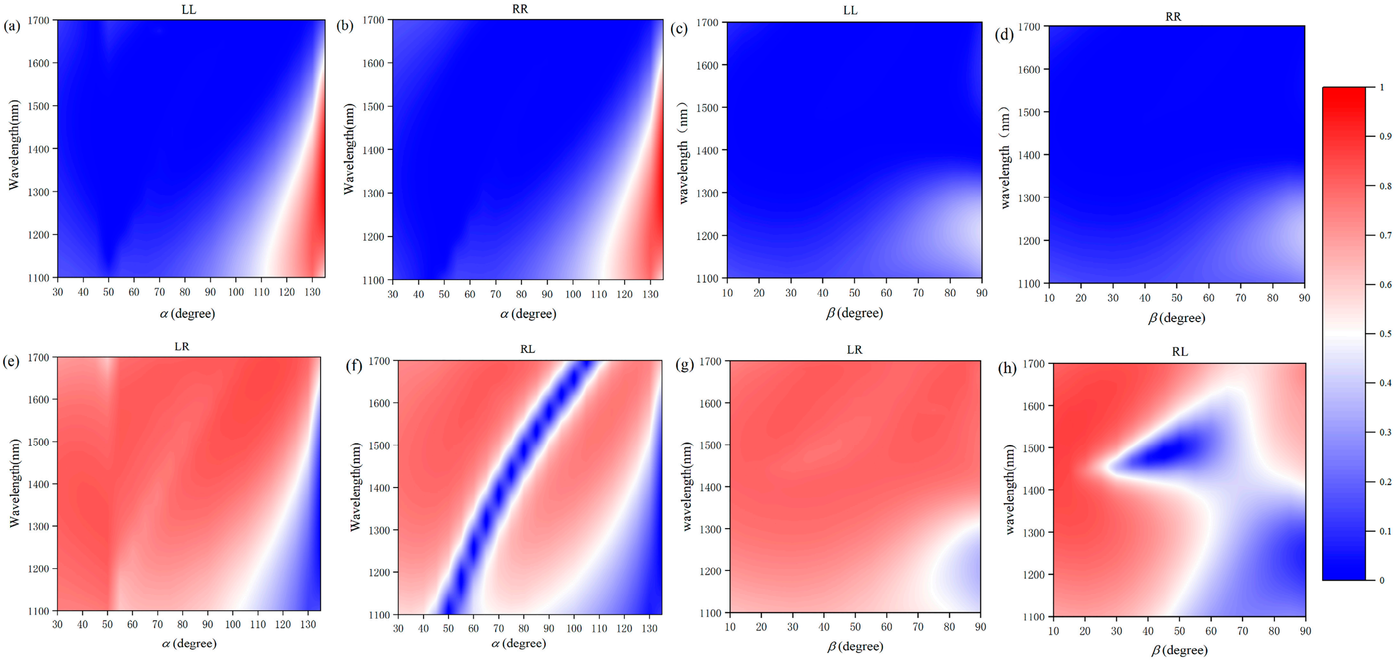

Figure S2. The effect of the α and β variation on the reflection intensity of the proposed chiral metasurface. The results are obtained with structural variables P=650 nm, L=160 nm, W=70 nm, M=70 nm, H=70 nm, T_1=300 nm, and T_2=180 nm under normal incident illumination. (a-d) The reflection intensity of r_{LL} and r_{RR} (the co-polarized components) of the proposed chiral metasurface with the changing of α and β. (e-h) The reflection intensity of r_{LR} and r_{RL} (the cross-polarized components) of the proposed chiral metasurface with the changing of α and β.

Section S3. The simulated absorption spectra under LCP and RCP illumination at normal incidence.

In theory, the absorption can be calculated by $A(\omega)=1-T(\omega)- R(\omega)$, where $T(\omega)$ and $R(\omega)$ are the transmittance and reflectance of the metasurface, respectively. However, the proposed metasurface is a MIM design, the ground Al layer can be regarded as a substrate which can be enough to prevent the transmission, so the absorption can be simplified as $A(\omega)=1-R(\omega)$. **Figure S3.** Shows the calculated absorption spectra of the proposed chiral metasurface in the wavelength range from 1100 to 1700 nm. The results demonstrate that the absorption exceeds 80% under RCP illumination from 1400 to 1500 nm, and the absorption remains basically unchanged at around 20% under LCP illumination from 1100 to 1700 nm, which is well agreed with the results of the reflected spectra (seeing **Figure 2c.**). In other words, we can find that the proposed chiral metasurface shows an excellent capacity for spin-selective manipulation due to the higher absorption during the corresponding operating wavelength band.

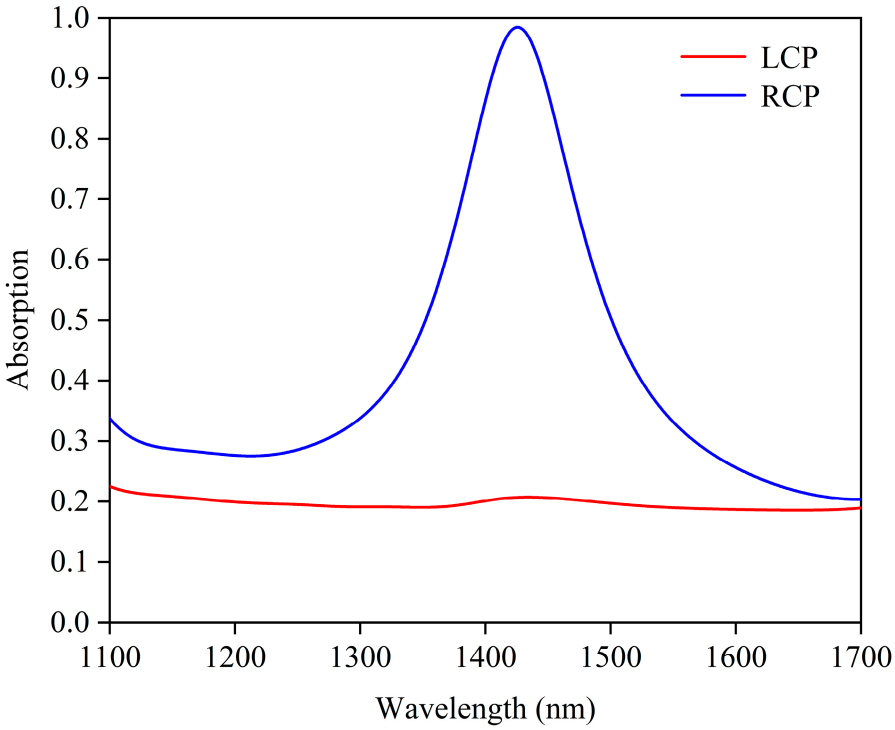

Figure S3. Simulated absorption spectra under LCP and RCP illumination from 1100 to 1700 nm.

Section S4. The electric field distributions on the *xoy* section at the wavelength of 1420 nm.

Further, we analyzed the electric field distributions on the *xoy* section under LCP and RCP illumination at the wavelength of 1420 nm, as shown in **Figure S4.** The results demonstrate that the E-field is concentrated at the ends of the long and short arcs under RCP illumination (seeing **Figure S4b.**), which is the main reason for the proposed chiral metasurface has a high absorption and an excellent capacity for spin-selective manipulation.

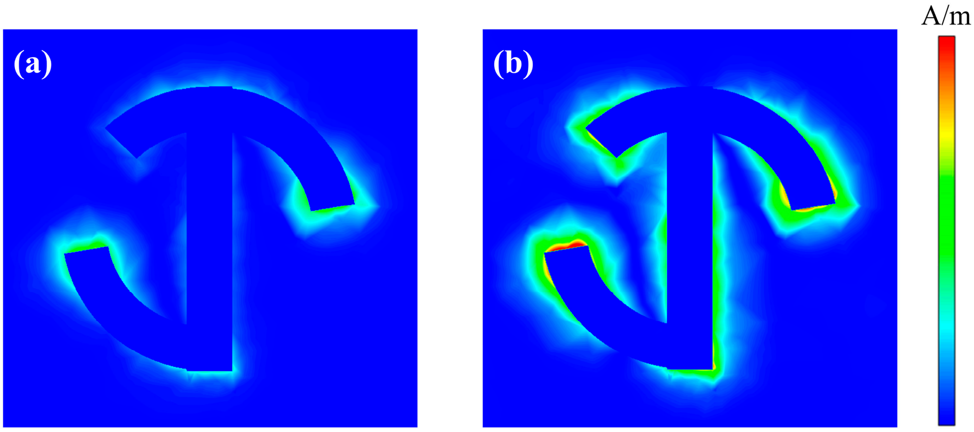

Figure S4. The electric field distributions on the *xoy* section at the wavelength of 1420 nm under (a) LCP illumination, (b) RCP illumination.

Section S5. The effect of incident angle variation on the reflection intensity of the proposed chiral metasurface.

To verify the effect of incident angle on the optical response of the proposed chiral metasurface, we studied the incident angle variation on the reflection intensity of the proposed chiral metasurface**. Figure S5 (a, b)** show that the reflection intensity of the co-polarized component is relatively low for whole angles. However, for cross-polarized

component, the proposed chiral metasurface still remains an excellent spin-selective manipulation when the incident angle is less 30°, as shown in **Figure S5 (c, d)**.

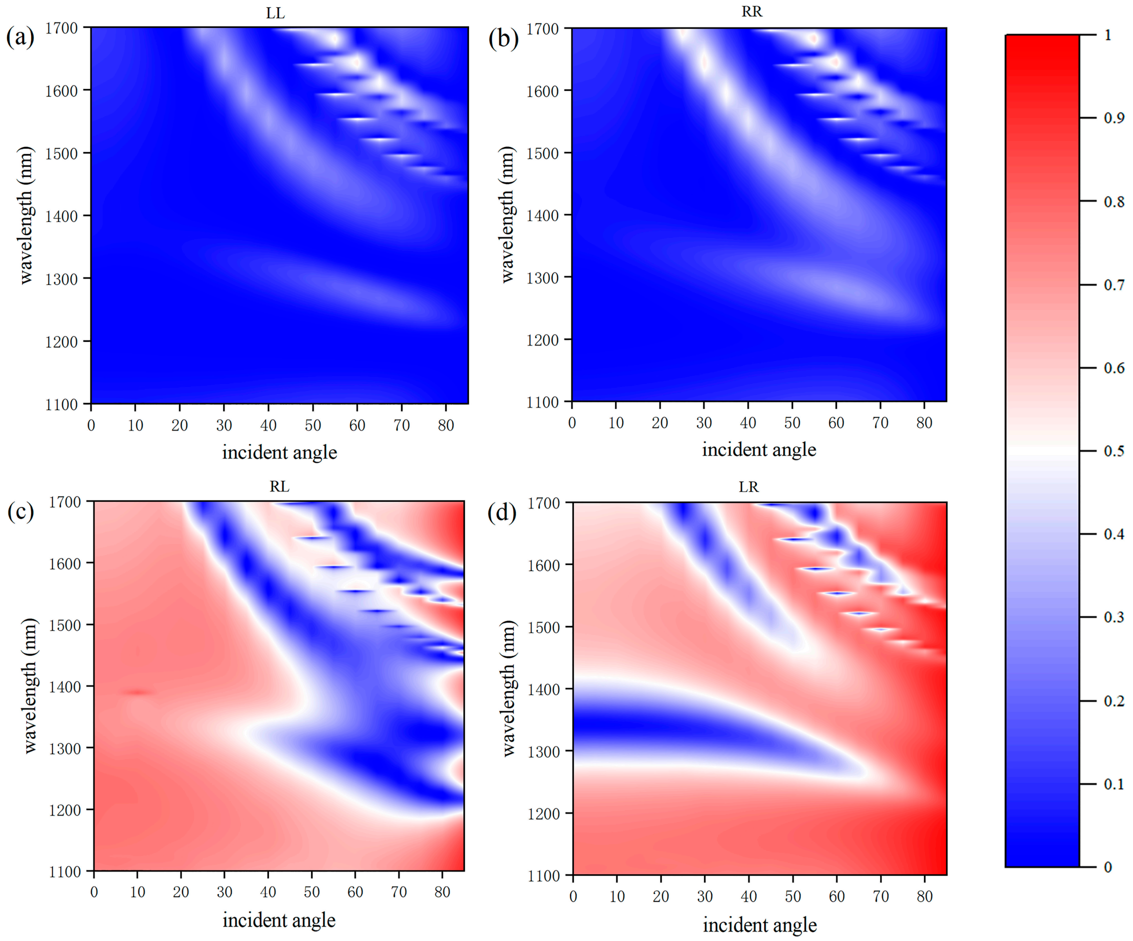

Figure S5. The effect of incident angle variation on the reflection intensity of the proposed chiral metasurface for different polarized components (a) is LL, (b) is RR, (c) is RL, (d) is LR.

Disclaimer/Publisher's Note: The statements, opinions and data contained in all publications are solely those of the individual author(s) and contributor(s) and not of MDPI and/or the editor(s). MDPI and/or the editor(s) disclaim responsibility for any injury to people or property resulting from any ideas, methods, instructions or products referred to in the content.
